# Long Term Copepod Culture Houses a Rich Microbial Eukaryotic Community Including New and Known Symbionts

**DOI:** 10.1111/jeu.70053

**Published:** 2025-11-03

**Authors:** Lasse K. Eliassen, Dag Altin, Tom Andersen, Lasse Riemann, Micah Dunthorn, Josefin Titelman

**Affiliations:** ^1^ Department of Biosciences University of Oslo Oslo Norway; ^2^ Research Infrastructure SeaLab Norwegian University of Science and Technology Trondheim Norway; ^3^ BioTrix Trondheim Norway; ^4^ Department of Biology University of Copenhagen Copenhagen Denmark; ^5^ Natural History Museum University of Oslo Oslo Norway

**Keywords:** anti metazoa, eukaryome, metabarcoding, parasitism

## Abstract

Copepods, dominant marine zooplankton, are hosts to microbial eukaryotic symbionts, but the copepod eukaryome remains largely unexplored. We used 18S rRNA gene primers with reduced metazoan amplification to identify microbial eukaryotes in a culture of *Calanus finmarchicus* (Copepoda). Samples were taken from the inlet water (99.5% of reads from non‐copepod sources) and the contents of the culture, which included ambient water (99.7%), bulk (many crushed copepods, 60.2%), individual copepods (1%–41%, mean = 7.4), and bulk fecal pellets (74%). The microbial eukaryotic community in the culture differed from the inlet water. The culture contained saprotrophs and bacterivores typical of eutrophic aquacultures and known parasites of copepods. Individual copepod eukaryomes varied in richness (8–33 operational taxonomic units, mean = 16.1) and revealed variation in non‐copepod read yields related to specific taxa. Perkinsea, not previously reported in copepods, as well as Ascomycota and Basidiomycota (Fungi), formed the core eukaryome (found in > 90% of individuals), indicating potentially important symbiosis. The small eukaryome, relative to reported microbiomes in *C. finmarchicus* , suggests that ecological inferences from microbiomes, which largely address bacteria, are not readily applicable to the eukaryotic microbes. The study underpins the need for investigations of eukaryomes.

## 1 Introduction

Copepods are the most abundant metazoans on Earth and dominate marine zooplankton communities (Verity and Smetacek 1996). As grazers of phyto‐ and microzooplankton and prey to many animals, coupled with their diel vertical migratory behavior, they significantly impact the oceanic carbon cycle (Steinberg and Landry 2017). This has motivated a wealth of research on their ecological impact as grazers and prey. Symbiotic interactions, however, are largely overlooked in marine food web dynamics (Worden 2015), despite the consensus that symbionts, of which parasites have been given most attention, can affect host populations (Kimmerer and McKinnon 1990; Skovgaard and Saiz 2006; Burris and Dam 2014), with ecosystem‐level consequences (Lafferty et al. 2008; Lima‐Mendez et al. 2015).

Copepods form symbiotic associations with viruses, prokaryotes, microbial eukaryotes (single‐celled eukaryotes including fungi), as well as other metazoans (Bass et al. 2021). Research on the microbial eukaryotic symbionts of copepods has mainly involved visual inspection of conspicuous parasites, most of which were described over a century ago (Chatton 1920). Ectosymbiotic ciliates on the exoskeleton and endosymbiotic dinoflagellates in the gut or hemocoel are most frequently reported in the literature (Bass et al. 2021). Few quantitative studies exist (reviewed in Eliassen et al. 2024), where host effects include starvation, castration, increased mortality, and behavioral changes (Kimmerer and McKinnon 1990; Torgersen et al. 2002; Skovgaard et al. 2012; Fields et al. 2015). Such effects are detrimental, comparable to predation (Kimmerer and McKinnon 1990), and can regulate host populations (Garvang et al. 2024).

The parasites on copepods appear sporadically both in space and time, although some follow seasonal patterns (Skovgaard and Saiz 2006; Cleary et al. 2022). Hence, sampling for quantitative host effects has typically been opportunistic (e.g., Fields et al. 2015; Eliassen et al. 2024) and cultivation for experiments has been unsuccessful. Moreover, many parasites are challenging or impossible to detect visually, generating a significant bias towards conspicuous species or late‐stage infections (Skovgaard and Saiz 2006).

The “microbiome” encompasses microorganisms associated with a biome and includes disparate organisms such as viruses, archaea, bacteria, single‐celled and colonial microbial eukaryotes (“protists”), and fungi. Generally, the term has become synonymous with bacteria (del Campo et al. 2020). However, microbial eukaryotes are more morphologically and behaviorally diverse than bacteria, while bacteria have higher biochemical and metabolic diversity (Keeling and Campo 2017). In response, the term “eukaryome” is used to better address the functionally distinct microbial eukaryotes (Lukes et al. 2015; del Campo et al. 2020), which may be settled on (ecto) or inside (endo) their host.

Molecular detection using metabarcoding is an alternative to visual inspection of microbial eukaryotic symbionts. Metabarcoding has revolutionized our understanding of pelagic microbial eukaryotic diversity (Massana et al. 2014) and has considerably advanced our understanding of the bacterial associations with copepods (Moisander et al. 2015; Skovgaard et al. 2015; Dorosz et al. 2016; Shoemaker and Moisander 2017; Datta et al. 2018; Yeh et al. 2020). However, its application to eukaryomes has proven difficult due to co‐amplification of host DNA because of similar genetic signatures (del Campo et al. 2020; Keeling and Campo 2017). Consequently, few metabarcoding studies explicitly investigate the eukaryome of copepods, and many reports stem from diet studies where symbionts were found by chance (Table 1). Attempts at circumventing host amplification include the use of host‐blocking primers that bind to host DNA and arrest its amplification during PCR (e.g., Cleary et al. 2022) and primers incompatible with the host genetic signature, which limit amplification (e.g., Guo et al. 2012), but these require tailoring to each system. Alternative solutions are deep sequencing protocols that prioritize read abundance at the cost of read length and, consequently, taxonomic resolution (e.g., Flo et al. 2024).

**TABLE 1:** Overview of molecular studies reporting on microbial eukaryotic symbionts in copepods, arranged by year of publication.

Study	Target	18S rRNA region	Primers		
				Host reduction method	
				Incompatible	Blocking
Guo et al. 2012	Symbiont	—	**Non‐copepod18S**	✔	
Yi et al. 2014	Diet	—	Non‐copepod18S (Guo et al. 2012) with ciliate blocking primer **ciliate18Sblk1**	✔	✔
Durbin and Casas 2014	Diet	—	960F and 1200R (Gast et al. 2004) with blocking primer **Cal‐PNA5**		✔
Ray et al. 2016	Diet	V7	F‐1183mod and R‐1443mod (Hadziavdic et al. 2014) with blocking primers **Cal‐SpcC3‐block** and **Cal‐PNA‐block**		✔
Cleary and Durbin 2016	Diet	V7	960F and 1200R (Gast et al. 2004) with blocking primer Cal‐PNA5 (Durbin and Casas 2014)		✔
Ho et al. 2017	Diet	V4	528F and 706R (Elwood et al. 1985) with PNA‐blocking primer **18s_cal_706r**		✔
Yi et al. 2017	Diet	—	Non‐copepod18S (Guo et al. 2012) with ciliate blocking primer ciliate18Sblk1 (Yi et al. 2014) and ciliate primers **Vampe18S**	✔	✔
Hirai et al. 2018	Symbionts	V9	1389F and 1510R (Amaral‐Zettler et al. 2009).		
Yeh et al. 2020	Diet	V4	Reuk454FWD1 and ReukREV3 (Stoeck et al. 2010)		
Zamora‐Terol et al. 2021	Symbionts	V4	528F and 706R (Elwood et al. 1985)		
Cleary et al. 2022	Symbionts	V7	960F and 1200R (Gast et al. 2004) with blocking primer Cal‐PNA5 (Durbin and Casas 2014)		✔
Holt et al. 2022	Symbionts	V4	18S‐EUK581‐F and 18S‐EUK1134‐R (Bower et al. 2004; del Campo et al. 2019)	✔	
Savage et al. 2023	Symbionts	V4	18S‐EUK581‐F and 18S‐EUK1134‐R (Bower et al. 2004; del Campo et al. 2019)	✔	
Serandour et al. 2023	Diet	V4	Nested PCR using non‐copepod18S Guo et al. (2012), then 528F and 706R (Elwood et al. 1985)	✔	
Flo et al. 2024	Diet	V7	18S allshorts (Guardiola et al. 2015)		
*This study*	Symbionts	V4	One‐step PCR (Minardi et al. 2021) using 574*F (Hugerth et al. 2014) and UnonMet_DB (Bass and del Campo 2020)	✔	

*Note:* The target column shows whether the study targeted symbionts or diet of copepods. The primers column shows the primers used in the respective studies and their source. Primers in bold were developed in the particular study (“Study” column). “Incompatible” refers to primers that reduce host amplification by being incompatible with the host's genetic signature. “Blocking” refers to oligonucleotides that bind to host DNA and thereby arrest its amplification.

Overall, many PCR protocols have been developed (Table 1), each with different biases, which hampers ecological inferences across studies. A more universal solution is primers that are incompatible across most metazoan hosts, that is, “anti‐metazoan”, such as the primers UNonMet_DB (Bower et al. 2004; Bass and del Campo 2020). The UNonMet_DB primers were successfully applied to copepods using a conventional two‐step PCR protocol (Savage et al. 2023). However, Minardi et al. (2021) achieved better performance and taxonomic coverage using a one‐step instead of a two‐step PCR protocol on various metazoans, which was tested on copepods for the first time in this study.

Establishing a way to study the eukaryome of copepods under controlled settings is an important pre‐requisite to studying eukaryome dynamics (Garvang et al. 2024, submitted; Keeling and Campo 2017), but attempts at experimental infections have been unsuccessful (cf. Chatton 1920; Kimmerer and McKinnon 1990; Skovgaard 2005). At NTNU SeaLab, Norway, *Calanus finmarchicus* has been kept in culture for over 80 generations (Hansen et al. 2007). *C. finmarchicus* is an ecologically important species in the North Atlantic Ocean (Planque and Batten 2000) and one of the better‐studied species in terms of microbial eukaryotic symbionts (Bass et al. 2021).

Here, we describe the microbial eukaryotes associated with the culture and its main constituent, *C. finmarchicus* , using the one‐step PCR metabarcoding protocol by Minardi et al. (2021). Our aims were to (i) explore which, if any, microbial eukaryotic symbionts of copepods are present in the culture and (ii) test the applicability of the metabarcoding protocol to describe copepod eukaryomes. These findings may provide a basis for future studies of eukaryome dynamics under controlled settings.

## 2 Materials and Methods

### 2.1 The *C. finmarchicus* Culture

The *C. finmarchicus* culture at NTNU Sealab, Trondheim, Norway, was established in 2004 from copepodite stage V collected in the Trondheimsfjord, Norway. The culture is supplied with seawater from 70 m depth, which runs through a sand filter and a holding tank with an average retention time of 36 h (5400 L volume, 150 L h^−1^ new water). Before entering the culture, the water is filtered for particles > 25 μm by cartridge filters. The culture facility holds multiple 250 L polyester tanks, each with 8–10,000 copepods (32–40 individuals L^−1^). The cultures are maintained at 8°C–10°C in a 16:8 light: dark cycle (2.0 μmol photons m^−2^ s^−1^, 400–700 nm), similar to the irradiance near the surface during April in Trondheim (63.4305°N) (Miljeteig et al. 2014). The water is exchanged at a rate equal to the volume of the tank per day (250 L d^−1^). The copepods are continuously fed *ad libitum* with a mixture of the microalgae *Rhodomonas baltica* and *Dunaliella tertiolecta* at a concentration above 150 μg C L^−1^ to support normal growth and development (Campbell et al. 2001). The algae source cultures are supplied with autoclaved seawater. The production cultures are supplied with seawater disinfected with sodium hypochlorite, NaOCl, and subsequent neutralization of excess chlorine, using sodium thiosulfate, Na_2_S_2_O_3_. New cultures are established from eggs from the previous generation, such that the age is homogenous. Each generation lasts ca. 75–90 days. There is no additional aeration or mixing of the water; hence particulate organic matter settles on the bottom.

### 2.2 Sampling

The culture contained adults at the time of sampling. Five sample types were collected from one of the culture tanks: inlet water (water before entering the culture, after the 20 μm cartridge filter), ambient water in the culture tank, a bulk sample of at least 5000 copepods, 30 individual C6 female copepods, and bulk fecal pellets. The gut content was not evacuated before collection.

To isolate the fecal pellet sample from the organic detritus in the culture, a subset of animals was transferred to a clean, 50 L tank with otherwise identical conditions, and fecal pellets were collected daily over 3 days. The collection was done by reducing the water in the tank to ca. 3 L by siphoning. The remaining water was screened over a 300 μm sieve to separate the animals before concentrating the fecal pellets on a 38 μm filter. The tank was rinsed with filtered seawater to remove any leftover fecal pellets before refilling and reintroducing the animals.

The inlet and ambient water samples, three replicates of 4 L each, were collected on 0.22 μm Sterivex GP Pressure filters (SVGV010RS, Sigma‐Aldrich, Missouri, USA) and stored at −20°C. The bulk copepod sample was collected in a 15 mL tube, from which three technical replicates containing 50%, 25%, and 25% of the animals, respectively, were made. The bulk sample was rinsed with sterile seawater on a 300 μm sieve followed by de‐ionized water on a 64 μm sieve. Excess water was removed with tissue paper, and the sample was fixed in 70% ethanol. The individual copepods were rinsed and fixed in the same way in separate 1.5 mL tubes. The fecal pellets were rinsed in sterile seawater and de‐ionized water, and excess water was removed before fixation in 70% ethanol in 1.5 mL tubes. The samples were shipped on dry ice from the culture facility in Trondheim to the University of Oslo for further processing.

### 2.3 DNA Extraction

Genomic DNA was extracted using the DNeasy Blood and Tissue kit (QIAGEN, Hilden, Germany), following the manufacturer's instructions. To prepare for extraction, bulk copepod and fecal pellet samples were homogenized using a Precellys 24 bead beater (Bertin instruments, Montigny‐le‐Brettoneux, France) (3 × 10 s, 5000 rpm) with 0.5 g of 1.4 mm diameter zirconium oxide beads, from which sub samples were taken for extraction. The individual copepod samples were similarly homogenized but in the lysis buffer provided in the extraction kit. For the water samples, the filter was removed from the plastic casing, frozen with liquid nitrogen, homogenized with a mortar and pestle, and added to tubes containing lysis buffer. Additional centrifugal rounds were needed during the extraction of the second and third bulk samples, suggesting that too much biomass was added. The inlet and ambient water sample extraction replicates were pooled, respectively, and each represents 12 L of water.

### 2.4 Library Preparation

A one‐step PCR protocol was used to produce dual‐indexed amplicons of the V4 region of the 18S ribosomal RNA gene with primers designed to amplify eukaryotes but exclude metazoans (Bower et al. 2004; del Campo et al. 2019; Bass and del Campo 2020). The one‐step protocol was chosen as it, in some cases, produces significantly fewer metazoan reads than the two‐step approach (Minardi et al. 2021). The primer combination used was the forward primer 574*F (5′‐CGGTAAYTCCAGCTCYV‐3′) by Hugerth et al. (2014) and the reverse, anti‐metazoan primer UnonMet_DB (5′‐CTTTAARTTTCASYCTTGCG‐3′) by Bass and del Campo (2020) with the Illumina adapter, index, pad+link, and primer, for example, [AATGATACGGCGACCACCGAGATCTACAC][ACGACGTG][TATCGCCGTTCG][CGGTAAYTCCAGCTCYV].

PCR reaction mixes were run in triplicates and comprised 1.8 μL of genomic DNA extract, 0.9 μL of each primer (10 μM), 9 μL Q5 High‐Fidelity 2X Master Mix (New England Biolabs, Massachusetts, USA), and 5.4 μL water to a final volume of 18 μL. The PCR thermal program consisted of an initial denaturation step of 5 min at 95°C, followed by 42 cycles of 98°C denaturation for 10 s, annealing of primers at 56°C for 30 s, and elongation at 72°C for 1 min, with a final elongation at 72°C for 5 min. Triplicates were pooled, cleaned using AMPure XP bead‐based reagent (Beckman Coulter, California, USA), quantified using Quant‐iT PicoGreen dsDNA Assay Kit (Invitrogen, Massachusetts, USA), and pooled in equimolar amounts. 300 bp paired‐end sequencing spiked with 15% PhiX was performed with an Illumina MiSeq (Illumina Inc., California, USA) instrument by the Norwegian Sequencing Centre at the Oslo University Hospital.

### 2.5 Bioinformatics

We used the swarm‐based pipeline by Mahé (2023a). In short, *Cutadapt* v4.9 (Martin 2011) was used to remove primers. *VSEARCH* v2.28.1 (Rognes et al. 2016) was used to merge and dereplicate the sequences, and *Swarm* v3.1.5 (Mahé et al. 2021) was used to generate molecular operational taxonomic unit (OTU) clusters with options *d* = 1 + fastidious. Hence, *Swarm* defines OTUs based on differences down to a single nucleotide (*d* = 1), rather than a specific cut‐off, and grafts low‐abundant OTUs onto higher‐abundant OTUs (fastidious). *VSEARCH* was used to remove chimeras and assign taxonomy using the *Protist Ribosomal Reference database* (PR2) (Guillou et al. 2013) v5.0. The associated PR2_traits database, with manual curation based on Bass et al. (2021) was used to assign trophic roles. The post‐clustering algorithm *Mumu* v1.0.2 (Mahé 2023b), which implements the *LULU* algorithm by Frøslev et al. (2017), was applied to the resulting OTU table, which combined OTUs based on sequence similarity and co‐occurrence patterns, using the settings minimum match 95, minimum_relative_cooccurence 0.01, and minimum_ratio 0.01. This reduced the number of OTUs from 7585 to 3337.

**FIGURE 1:**
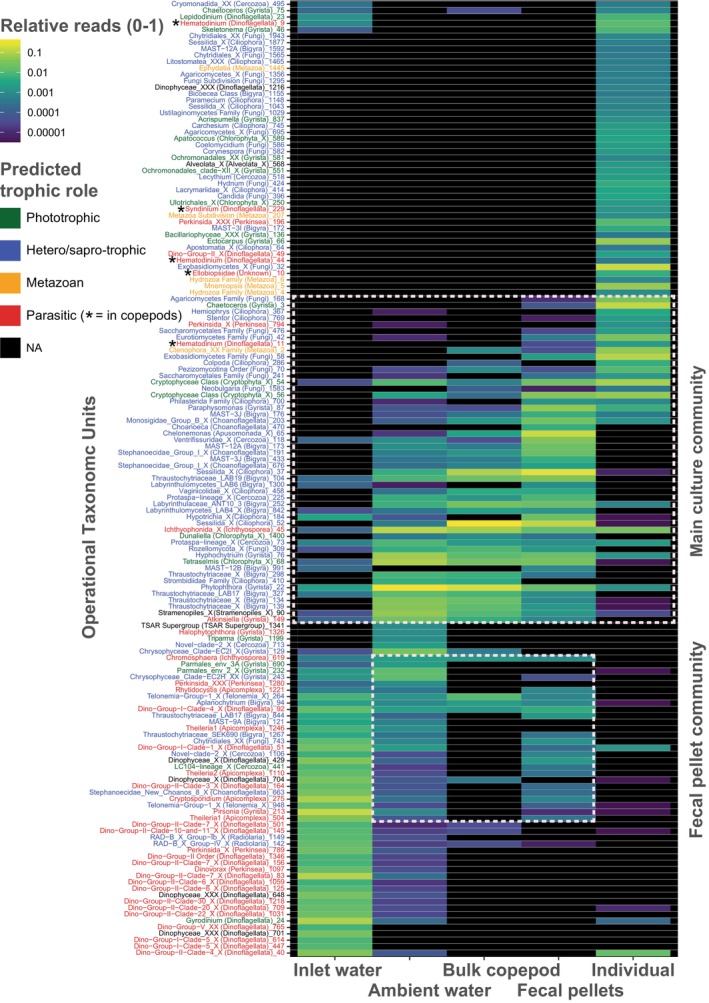
Heatmap of the Operational Taxonomic Units (OTUs) detected in the samples (replicates and individual copepods were normalized and averaged), using the plot_heatmap function in the phyloseq R package (method = “NMDS”, distance = “bray”). The OTUs are ordered by the first ordination axis, while the trophic role color coding is assigned by PR2_traits (see methods). The white, dotted boxes and descriptions on the right‐hand side highlight the main culture and fecal pellet communities. Black indicates no detection.

**FIGURE 2:**
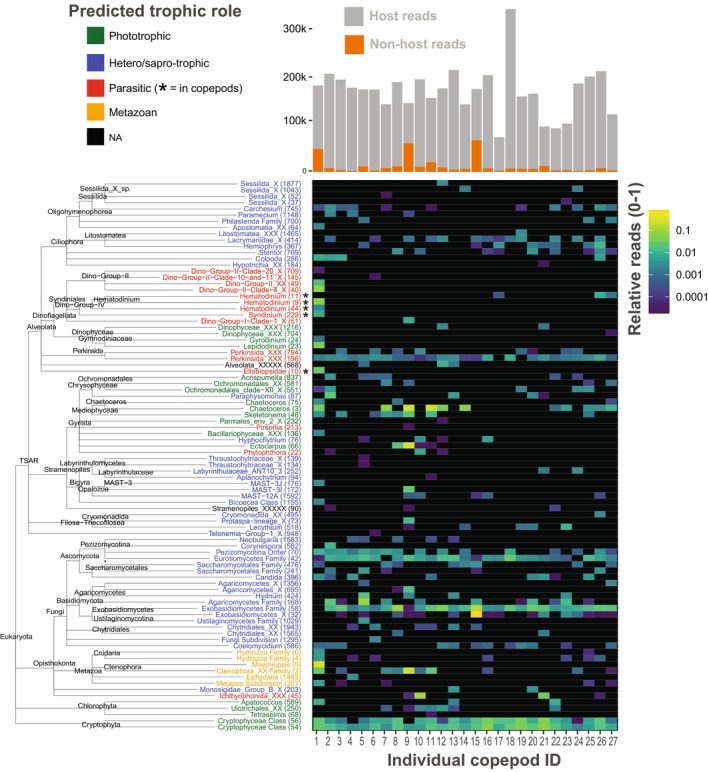
Heatmap of the Operational Taxonomic Units (OTUs) in the individual copepods (*n* = 27). The OTUs are ordered according to their taxonomy (left) and individual are ordered based on ordination using the plot_heatmap function in the phyloseq R package (method = “NMDS”, distance = “bray”). A taxonomic assignment of X means that the closest assignment was the nearest node in the tree. Black indicates no detection. The trophic role color coding is assigned by PR2_traits (see methods). The bar plot above the heatmap shows the absolute number of reads from host (gray) and non‐host sources (orange).

In total, 4,969,857 reads were obtained, of which 722,227 (14.5%) reads from 149 OTUs remained after filtering out host sequences (Arthropoda) and subsequently rare OTUs. Rare OTUs were defined as those contributing < 1% in the cumulative read count distribution of the dataset. The proportion of reads and OTUs remaining after the two filtering steps relative to the raw data is referred to as “final yield”. The latter step reduced the number of OTUs without affecting the overall compositional patterns. Analyses and figures were done in R v4.3.1 (R Core Team 2023). The plot_heatmap function in the phyloseq package (McMurdie and Holmes 2013) was used to plot the heatmaps in Figures 1 and 2.

## 3 Results

Despite the equimolar pooling, the total number of reads from the inlet and bulk copepod samples was low (22,930 and 36,138 reads per sample, respectively) and could have benefitted from deeper sequencing although final yields were high (Table 2). The first bulk replicate (50% of biomass) had a final yield of 60.1%, whereas the second and third bulk replicates (25% each) had a lower combined final yield of 2.7% (3.1 and 2.3, respectively). The second and third bulk replicates both required additional centrifugal steps during DNA extraction, suggesting that the spin column was overloaded. When spin columns are overloaded, the most abundant DNA, that is, copepod DNA, is likely to saturate the membrane, and rarer DNA may be lost, which may explain the reduced final yields in the second and third bulk replicates (Table 2). The final yields in the individual copepod ranged from < 1 to 41%, with a mean of 7.3 ± 10 (mean ± SD, *n* = 27). The number of OTUs per individual ranged from 8 to 33 with a mean of 16.1 ± 4.9 (*n* = 27).

**TABLE 2:** Overview of samples showing the combined reads and Operational Taxonomic Units (OTUs) remaining after filtering out host reads (Copepoda) and subsequently rare OTUs (defined as those contributing < 1% of the cumulative read count).

	Inlet water	Ambient water	Bulk copepod		Fecal pellets	Individual copepods
Biological replicates	3	3	3		3	27
Sequencing replicates	1	1	1^a^	2	3	27
Raw reads	22,930	191,223	36,138	136,657	229,680	4,352,631
After removing host	22,808	190,958	21,771	24,344	169,973	320,356
%	**99.5**	**99.7**	**60.2**	**17.8**	**74.0**	**7.4**
After removing rare OTUs	20,155	190,641	21,691	3746	168,753	317,236
%	**87.9**	**99.7**	**60.1**	**2.7**	**73.5**	**7.3**
Raw OTUs	152	130	50	45	107	165
After removing host	151	129	44	31	95	147
%	**99.3**	**99.2**	**88.0**	**68.9**	**88.8**	**89.1**
After removing rare OTUs	68	88	38	27	68	88
%	**44.7**	**67.7**	**76.0**	**60.0**	**63.6**	**53.3**

*Note:* Numbers in bold indicate the percentage of reads/OTUs relative to the raw data.

^a^ Bulk replicate #1 is presented separately to #2 and #3 (combined) to highlight the variation.

### 3.1 Taxonomic Composition in the Culture and Individuals

The community of microbial eukaryotes in the culture (i.e., ambient water), bulk copepods, fecal pellets, and individuals differed from the inlet water, which had many OTUs from the parasitic group Syndiniales (Figure 1). Six photosynthetic OTUs were detected in the inlet water. Most of the inlet water taxa were also detected in the ambient water. The ambient water and fecal pellets shared taxa not found in the copepods, suggesting that there is a community of microbial eukaryotes in the culture reliant on the fecal pellets. The ambient water, bulk copepod, and fecal pellets formed a group that shared taxa mainly from Bigyra and Fungi (Figure 1, middle). The most common trophic roles in this community were hetero‐ and saprotrophy. Many taxa were detected in the individual copepod samples and not the bulk copepod sample (Figure 1, top), although this is likely a consequence of the greater collective sequencing depth of these 27 individuals (317,236 reads) relative to the bulk copepod samples (25,437) (Table 2). Several photosynthesizing taxa, primarily from Gyrista, were detected in the culture, in addition to the supplied diet of *Dunaliella tertiolecta* (Chlorophyta) and *Rhodomonas baltica* (Cryptista).

Groups commonly associated with copepods found in the culture included Ciliophora, Syndiniales (*Syndinium* and *Hematodinium*), and Ellobiopsidae (Figures 1 and 2). The latter two are definite parasites of copepods, whereas the ciliates for example, the sessilid *Carchesium*, found in 11 out of 27 individuals, could be symbionts or free‐living in the culture and thus prey of *C. finmarchicus* (Calbet and Saiz 2005; Boscaro et al. 2018). Apicomplexans were also present in the culture and are known to infect copepods (Bass et al. 2021), but were not detected in the bulk or individual samples. Prevalent groups detected in the individuals included the parasitic Perkinsea (found in 26/27 individuals) and the two representatives of the fungal groups Ascomycota (25/27) and Basidiomycota (26/27) (Figure 2). Other parasitic groups detected were Ichthyophonida (Ichthyosporea), MAST‐3 (Bigyra), and *Pirsonia*, *Hypchoctrium*, *Phytophtora* (Gyrista). The diet of *R. baltica* (OTU 54 and 56) (Cryptophyta) had a strong signal in the individuals, while *D. tertiolecta* (Chlorophyta) was not found, despite detection in the ambient water, bulk, and fecal pellets (Figure 1, OTU 1400). Photosynthesizing taxa from Chrysophyceae and Mediophyceae (Gyrista), particularly *Chaetoceros* and *Skeletonema*, suggest that the culture sustains phototrophs that are potentially grazed upon by *C. finmarchicus* . Five metazoan genera were also detected in the individuals, with Ctenophora being present in 10 out of 27 individuals (Figure 2), despite being absent in the inlet and ambient water (Figure 1).

Some individuals had a greater non‐copepod read yield (Figure 2, top), for example, individuals 1, 9, and 15, which had yields of 26%, 38%, and 41%, respectively. These individuals also had a distinct symbiont profile, including the presence of several Syndiniales and metazoan OTUs, as well as the only instance of Ellobiopsidae. The connection between yield and taxa suggests that these specific individuals may have been more heavily burdened by symbionts than the others.

## 4 Discussion

### 4.1 Known and New Symbionts of Copepods

Known and potential symbionts from several taxa were detected in the culture and individual copepods (Figures 1 and 2). Except for certain confirmed copepod‐associated lineages, for example, *Ellobiopsis* and *Syndinium*, the nature of the relationship between the detected organisms and the copepods, or their ecology in the culture, is cautiously discussed based on available knowledge of the groups and their detection patterns. Some might not be symbionts of *C. finmarchicus* per se, but instead may interact within local food webs around the fecal pellets or the bottom. For example, *Phytophtora and Pirsonia*, found in the culture, parasitize marine plants and diatoms (Kühn et al. 2004; Meng et al. 2014). This also applies to Ichthyosporea, a parasitic group that causes heavy losses in pelagic fisheries (Kocan 2019). Ichthyosporea is also found in *Daphnia* (Lohr et al. 2010) but has not been confirmed in copepods. Although Ichthyosporea was prevalent in the culture, it was also present in the inlet water, raising doubt as to its specific interaction with *C. finmarchicus* . Similarly, apicomplexans, known to parasitize copepods, were present in the culture but not in the copepod samples (bulk or individuals).

Perkinsea is likely a core symbiont of *C. finmarchicus* in the culture, as is probably the fungi Eurotiomycetes (Ascomycota) and Exobasidiomycetes (Basidiomycota) based on their presence in > 90% of the individuals (Figure 2). The established parasites of copepods, Syndiniales (*Hematodinium* and *Syndinium*) and Ellobiopsidae, appear to associate more sporadically. Cultivating microbial eukaryotic symbionts of copepods has been a long‐standing challenge (Chatton 1920; Kimmerer and McKinnon 1990; Skovgaard 2005). Given the obstacles of entering the culture from the outside, it is possible that some of the symbionts, particularly Perkinsea, complete their life cycle in the culture (but see for example *Hematodinium*, Figure 1, top). If so, these symbionts represent possible candidates for further studies of symbiont–copepod dynamics *ex situ*, an important milestone in this field.

Perkinsea is a diverse group of intracellular parasites not previously associated with copepods. They are found in both freshwater and marine ecosystems and infect a diverse range of taxa including dinoflagellates, bivalves, frogs, and fish, and typically cause large population declines (Alacid et al. 2020; Metz et al. 2023). The OTU detected in this study was most closely related to a sequence from a freshwater pond in the United Kingdom, which clusters within the NAG01 group (96% sequence similarity: Metz et al. 2023). The NAG01 group is primarily associated with freshwater and amphibians; hence, our finding sheds new light on the habitat and host range of Perkinsea, which currently consists mostly of undescribed diversity (Metz et al. 2023).

Fungi are common in copepods and are considered both mutualists and parasites (Feng et al. 2023). Given their role as saprotrophs, the nutrient‐rich, high‐density culture systems may provide better conditions for saprotrophs than natural plankton communities (Duffy et al. 2012). However, both Eurotiomycetes (OTU 42) and Exobasidiomycetes (OTU 58), the two prevailing fungal groups in this study, are common in a range of arthropod hosts (Holt et al. 2022). Hence, their ubiquitous presence in the individual copepods may indicate a symbiotic relationship, suggesting that copepods rely on fungi, possibly for nutrient recycling, or *vice versa* (Poulsen and Boomsma 2005; Feng et al. 2023). As the closest hit to OTU 58 was *Malassezia*, a fungal group that utilizes exogenous lipids (Juntachai et al. 2009), the fungi may also be parasitic on lipid‐rich calanoids like *C. finmarchicus* (Eliassen et al. 2024). Indeed, *Malassezia* has also been detected in another calanoid, *Calanus sinicus* (Yi et al. 2017).

Although not expected, Ctenophora and Hydrozoa were detected in several individuals, and Ctenophora was also detected in the bulk sample (Figure 2). They appear in metabarcoding studies of copepods, where they are considered part of the diet (e.g., Cleary et al. 2022; Savage et al. 2023). However, both groups encompass parasitic lineages, and some hydrozoans are confirmed parasites of copepods (Haddock 2007; Bass et al. 2021). It is thus conceivable that the OTUs represent symbionts rather than ingested prey, particularly since they were absent in the ambient water (cf. Figure 1).

### 4.2 Diversity of Microbial Eukaryotes

The community in the culture was rich and distinct from the inlet water (Figure 1, middle) and had a trophic profile consisting of consumers of small algae or bacteria, saprotrophs, and some parasites. In addition, some phototrophs live in the culture, most notably *Chaetoceros*. The culture is minimally disturbed with a water exchange rate of 1 tank per day (250 L^−1^ d^−1^/250 L), and conditions along the walls and bottom of the culture resemble those of a eutrophic lake with detritus and algal mats. Hence, the waste generated by the animals and the supplied diet, such as fecal pellets, carcasses, eggs, and resting spores (Møller et al. 2003; Poulsen and Kiørboe 2006; Iversen and Poulsen 2007; Tang and Elliot 2014; Rodríguez‐Martínez et al. 2020), facilitate a community of bacterivores and detrivores, as is the case in other aquacultures (Zheng et al. 2021). Indeed, there was a community of consumers, but also parasites, found in the fecal pellets and ambient water but not in the copepods (Figure 1).

*C. finmarchicus* grazes most efficiently on the pelagic community of the culture. Assuming a clearance rate of 0.6 L d^−1^ per individual (Kiørboe and Hirst 2014) and a density of 40 individuals L ^−1^, *C. finmarchicus* can clear the culture ca. 25 times per day. Hence, the microbial eukaryotic community in the culture is likely the product of both the top‐down control of *C. finmarchicus* feeding and the bottom‐up facilitation by the organic matter.

### 4.3 Metabarcoding, Prevalence Estimates, and Disease Burden

This is the first application of the one‐step anti‐metazoan protocol on copepods and among few to report the eukaryome of individual copepods (see Hirai et al. 2018). Non‐host read yields were high in the water, bulk copepod, and fecal pellet samples (Table 2). They were lower in the individuals, with a mean of 7.3% (Table 2), although 60% higher than Hirai et al. (2018) who studied the gut contents of *Calanus sinicus* . Minardi et al. (2021), who developed the protocol used in this study, also found substantial variation in performance between sample types, with metazoan reads varying from 92% in common prawn to 4.8% in whiteleg shrimp (see table 1 in Minardi et al. 2021). Of the reads from the 27 individual copepods in this study, 59% came from three individuals: 1, 9, and 15 (Figure 2). These three individuals, particularly 1, stood out from the rest with several unique taxa, including metazoans and syndinids. Excluding these, the yield was 4.2% ± 3.1% (± SD, *n* = 24). Despite the low yields in the remaining individuals, clear patterns with various taxa were observed (Figure 2B).

Our findings have implications for prevalence estimates of symbionts. Prevalences based on visual inspection in the field are often lower than 0.001%, although peaks of much higher prevalence occur (Kimmerer and McKinnon 1990; Skovgaard and Saiz 2006; Coats et al. 2008; Alves‐de‐Souza et al. 2011; Fields et al. 2015). With less than 30 individuals, we detected several symbionts, including multiple instances of *Hematodinium* (Figure 2). This high occurrence suggests the culture may be conducive to infection, possibly related to the density of *C. finmarchicus* in the culture, which is two orders of magnitude greater than the maximum recorded in large‐scale surveys of North Sea surface waters (40 vs. 0.2 copepods L^−1^) (Heath et al. 2008), although rare patches of up to 100 individuals L^−1^ can occur (Parks et al. 2012).

It is also possible that we have detected early stages of the infection or ingested spores that do not result in infection. Visual inspection underrepresents endoparasites due to the difficulty in detecting the early stages of infection (Skovgaard and Saiz 2006). Our findings suggest that metabarcoding provides prevalence data beyond the capacity of visual inspection (Bass et al. 2015). Hence, metabarcoding may be an important tool for exploring ontogenic parasite–host relationships and for improving prevalence estimates, which are required for modeling the dynamics of parasite–copepod infections (Anderson and May 1981).

The same primers applied to other animals with large infections (Minardi et al. 2021) have potential for high non‐metazoan yields, supporting both the efficacy of the primers and the plausible relationship between yield and degree of infection. Under this assumption, our results suggest that most of the copepods in the culture have a minor presence of microbial eukaryotes. This harmonizes with general parasitology, where, although ubiquitous in most populations, parasites tend to aggregate in a small subset of individuals (McVinish and Lester 2020). We recognize that yield should be interpreted cautiously as read abundance does not necessarily reflect actual abundance or ecological impact (Deagle et al. 2019), and primers may be biased towards or fail to amplify certain taxa (Bass and del Campo 2020).

Datta et al. (2018) analyzed the bacterial microbiomes of 200 individuals of *C. finmarchicus* . They detected a core microbiome of 34 OTUs out of 241 OTUs (14%), defined as being present in > 90% of the individuals. Applying the same cut‐off to our data results in a core eukaryome of only three OTUs out of 88 (3.4%): Perkinsea, Ascomycota, and Basidiomycota (OTU 196, 42, and 58, respectively). The much smaller core eukaryome observed here may reflect inherent differences between microbial prokaryotes and eukaryotes such as size (Keeling and Campo 2017), particularly as copepods have sensory and tactile abilities for handling eukaryotic prey (Kiørboe 2013) that are similarly sized to the dispersal stages of microbial eukaryotic symbionts. Thus, lessons from the bacterial microbiome of copepods (e.g., Skovgaard et al. 2015; Moisander et al. 2015; Dorosz et al. 2016; Shoemaker and Moisander 2017; Datta et al. 2018; Yeh et al. 2020) may not be readily applicable to their eukaryome, necessitating a complementary, eukaryome‐driven approach.

### 4.4 Bulk Samples and the Sum of Individuals Are Not the Same

The bulk and individual copepod samples had few overlapping taxa (Figure 1). The higher number of OTUs in the individual samples (Figure 1, top right) likely results from the difference in sequencing depth (Table 2), but this should not affect the core patterns (Sato et al. 2017; Mata et al. 2018). While ciliates dominated the bulk samples, fungi dominated the individual samples. Plausibly, infected individuals in a pooled sample drive read patterns and reduce effective read depth available for other taxa. This becomes evident when pooling the 27 individuals in this study, where three individuals alone make up 59% of the non‐host reads. Alternatively, differences in biomass between the bulk and individual samples may have caused biases in DNA extraction (Wong et al. 2020) affecting yields (see Results). While most studies reporting microbial eukaryotes associated with copepods involve pooling (Table 1), our results suggest that pooled samples may poorly represent individual eukaryomes. This is particularly relevant for studies reporting species‐specific eukaryomes, which are likely to involve pooling of relatively few animals.

## 5 Conclusion

We detected microbial eukaryotes associated with a long‐term copepod culture using an anti‐metazoan metabarcoding protocol (Minardi et al. 2021). Our study revealed a community of microbial eukaryotes typical of marine cultures and several parasitic associations with *C. finmarchicus* . Some parasites may complete their life cycle in the culture and represent candidates for future studies under controlled settings. This is the first report of Perkinsea in copepods, which expands their eukaryome to include a large parasitic group that causes population declines in other hosts (Metz et al. 2023). Perkinsea, Ascomycota, and Basidiomycota formed a core eukaryome in the culture, significantly smaller than core bacterial microbiomes reported in *C. finmarchicus* (Datta et al. 2018). Microbial eukaryotes are considered part of microbiomes in most definitions, although microbiome studies often only address bacteria (Keeling and Campo 2017). The discrepancy in richness between the core biomes of microbial eukaryotes and bacteria suggests that ecological inferences from bacterial microbiomes may not be readily applicable to eukaryomes.

## Author Contributions

L.K.E., T.A., D.A. and J.T. designed the project. D.A. collected the samples from the culture. L.K.E. performed the molecular lab work. M.D. provided feedback on bioinformatics and taxonomy. L.K.E. drafted the paper, with inputs from T.A., J.T., D.A., L.R. and M.D.

## Conflicts of Interest

The authors declare no conflicts of interest.

## Data Availability

Data and scripts are available at Zenodo: https://doi.org/10.5281/zenodo.13767134.
